# Health problems among disaster responders to the 2023 Turkey-Syria earthquake: a cross-sectional study

**DOI:** 10.1186/s12873-024-01143-2

**Published:** 2024-12-02

**Authors:** Karin Hugelius, Jason Murphy, Karin Blomberg

**Affiliations:** 1 Faculty of Medicine and Health, Örebro university, Örebro, SE-701 82 Sweden; 2 Department of Surgical and Perioperative Sciences, Umeå University, Umeå, Sweden; 3 The Red Cross University College, Stockholm, Sweden

**Keywords:** Disaster, Disaster responder, Health, Mental health problem, Disaster response, Duty of care

## Abstract

**Objective:**

The aim of this study was to describe perceived health problems among disaster responders after the earthquake in eastern Turkey/Syria in February 2023.

**Methods:**

A non-probability cross-sectional study was conducted using an online survey.

**Results:**

A total of 525 local (18%) and international disaster responders (81%) participated in the study. Of these responders, 46% reported physical or mental health problems during or after their deployment, 15% required medical care during the mission, and 7% required medical evacuation. The most common health problems during the field mission were feeling scared or unsafe, sleeping problems, and headache. After the mission, fatigue, sleeping problems, and feeling depressed were the most frequently reported health problems. The local responders perceived significantly more health problems than did the international responders. Approximately 11% of the participants could not return to their ordinary work after deployment because of infections or mental health issues.

**Conclusions:**

Physical and mental health problems are commonly perceived by disaster responders and may reduce the effectiveness of disaster response. Raising awareness of health risks among disaster response workers and employers is essential to ensure proper duty of care and should include reparations and medical support during and after disaster response operations.

**Supplementary Information:**

The online version contains supplementary material available at 10.1186/s12873-024-01143-2.

## Introduction

Around 4:00 am on February 6, 2023, a 7.8-magnitude earthquake struck the southeast parts of Turkey and Syria (GLIDE Nos. EQ-2023-000015-TUR and EQ-2023-000015-SYR), followed by several strong aftershocks. One week later, 29,605 fatalities and 80,300 persons with injuries caused by the earthquake were reported in Turkey, and 5714 fatalities and 9949 persons with injuries were reported in Syria [1]. At the same time, urban search and rescue (USAR) teams from 83 countries, including approximately 10,423 personnel and 364 search dogs, were deployed to the area [2], as well as several emergency medical teams (EMT) and hundreds of humanitarian experts representing local responders, non-governmental organizations, United Nations (UN) agencies, and the Red Cross. Deployment to a disaster area after an earthquake is associated with certain risks. Both physical and mental health problems have been previously reported among disaster responders. In humanitarian missions by the International Red Cross, more than 80% of field staff reported health problems during their mission, most of which were related to diarrhea, fever, dental, skin, or musculoskeletal problems [3] and accidents, mainly sports or traffic accidents. Almost a third reported worsening health upon returning home compared with before deployment [3]. Voluntary responders to the earthquake in Nepal in 2015 reported gastrointestinal problems, skin problems, injury, and musculoskeletal problems, respiratory problems, syncope, and psychological problems [4]. In addition, USAR responders are exposed to risks related to working in confined spaces and rubble; inhalation of dust, asbestos, or carbon monoxide; biohazards from living and deceased victims; unsecured utilities such as natural gas or electricity; and explosive hazards such as gasoline [5].

In addition to physical health problems, disaster responders are also at risk of mental health problems that can negatively impact them during, upon their return to, and over a long time after their deployment [6]. Most research studies on the health outcomes of disaster responders have focused on mental health problems [7]. Among disaster responders, the prevalence rate of posttraumatic stress disorder (PTSD) has been reported to range from 0 to 34%, and that of depression ranges from 21 to 53% [7]. Being part of an international response operation such as that after the Turkey/Syria earthquake includes facing language and cultural barriers and massive human suffering [8, 9]. Disaster responders often underestimate these risks and their own well-being, focusing on their duty to help others [10]. However, it is important that disaster responders stay healthy and reduce their health risks, as medical incidents may reduce the possibility of delivering lifesaving and humanitarian support to those affected by the disaster, and not to add burden on the already strengthened health-care system and the response community [11]. Despite previous studies on the health effects of being deployed in the early aftermath of a disaster, such knowledge is still limited, and little is known about the problems that occur in different stages of the rescue mission and possible risk factors of serious medical incidents [10]. Such knowledge is essential in preparing disaster responders and reducing the risk for medical emergencies in the field and after the end of the mission [3, 8].

The aim of this study was to describe perceived health problems of disaster responders after the Eastern Turkey/Syria earthquake in February 2023.

## Methods

### Study design

A non-probability, cross-sectional study was conducted.

### Study sample and setting

After the earthquake, local and national resources were the first on-site, including professional and voluntary responders such as medical personnel, firefighters, military personnel, and Red Crescent volunteers. These were later followed by a massive international response that included over 10,000 USAR team members, UN agencies, and European Union (EU) civil protection teams [2]. No official information is available on the number of disaster responders involved. Therefore, an international nonprobability study sample was used.

The inclusion criteria for the study were participants actively involved in disaster response who were at least 18 years of age at the time of the incident and had the ability to respond to the questionnaire in either English or Turkish. Study invitations were sent out to organizations listed on official internet pages such as Reliefweb.int and to the International Federation of the Red Cross, Médecins Sans Frontières, Save the Children, United Nations Office for the Coordination of Humanitarian Affairs, World Food Program, UNICEF, World Health Organization EMT Secretariate, the international search and rescue advisory group secretariate, and local medical response teams, requesting their support to send the invitation to individuals who had been involved in response. The invitation was sent out 8 weeks after the earthquake and was open from March 24 to July 30, 2023.

### Questionnaire

A study-specific questionnaire was developed by the research team (see supplementary file). The questionnaire was strongly influenced by previous studies that were aimed at identifying health problems among first responders after disasters or mass casualty incidents [4, 7, 12]. The questionnaire consisted of multiple-choice questions on preparations before the mission, physical and mental health problems during or after the mission, and consequences of any reported health problems. The study participants were also asked to rate their overall perceived health using the EQ visual analog scale (EQ-VAS; marking from 0 to 100, where higher numeric scores represent better patient function). The questionnaire was available in English and Turkish, free of choice by the study participants. In addition to questions on perceived health problems, questions on perceived competence and preparation were also asked. The results of the questionnaire survey has been reported elsewhere [13]. The questionnaire was piloted among 10 Swedish disaster responders with experiences from several international operations (not including the earthquake in Turkey/Syria). Minor changes in the wording and layout of the questionnaire were made after the pilot test.

### Analysis

Descriptive and inferential statistical analyses were performed, including *χ*^2^ and logistic regression tests. A p value of ≥ 0.05 was considered statistically significant. Missing values were excluded from the analysis. The STROBE checklist was used to report the results. IBM SPSS Statistics for Windows, Version 28.0 (released 2021; IBM Corp., Armonk, NY) was used to analyze the data.

## Results

### Demographics

In total, 525 disaster responders were included in the study. Of these responders, 360 (69%) were male and 165 (31%) were female, with a mean (SD) age of 42 (10.5) years (range, 22–65 years). Of all the study participants, 81% (*n* = 423) were international responders and 93 (18%) were national responders. Most responders (*n* = 438, 83%) were deployed on a voluntary basis.

Nearly 55% (*n* = 288) of the participants had no previous experience from being deployed to a disaster area, 13% (*n* = 68) had experience from five or more missions, and 1% (*n* = 6) had been deployed to 15 missions or more. Most participants were deployed in the operation for up to 3 weeks (see Table 1).

**Table 1:** Demographics of the study sample (*N* = 525)

		Total *n* (%)
**Gender**	Male	360 (69%)
	Female	165 (31%)
**Marital status**	Married	312 (59%)
	Single	210 (40%)
**Have children**	Yes	252 (48%)
Profession in everyday life*	Physician	57 (11%)
	Nurse	117 (22%)
	Rescuer/firefighter	21 (4%)
	Military/police officer	39 (7%)
	Social worker/psychologist	36 (7%)
	Logistical officer	30 (6%)
	Information and communication technology officer	12 (2%)
	Humanitarian aid worker	78 (15%)
	Other	30 (12%)
**Local**,** regional**,** or international responder**	National responder	93 (18%)
	International responder	423 (81%)
**Type of deployment**	Voluntary	438 (83%)
	Mandatory	87 (17%)
**Type of response**	Emergency medical team (EMT)	108 (21%)
	Non-EMT health response	48 (9%)
	Urban search and rescue	123 (23%)
	Mental health and psychosocial support	36 (10%)
	Needs assessment	24 (5%)
	Management, coordination, and logistics	87 (17%)
	Shelter	18 (3%)
	Food or nutrition	21 (4%)
	Other	57(11%)
**Length of mission**	1 to 7 days	247 (47%)
	8 to 21 days	186 (35%)
	22 days or longer	93 (18%)

*Does not necessarily imply the position during the disaster response mission.

### Perceived health problems

In total, 244 study participants (46%) reported some type of health problem related to the mission. Study participants involved in mental health and psychosocial support response reported most health problems (*n* = 27, 75%), followed by participants involved in needs assessment (*n* = 16, 67%) and water and sanitation response (*n* = 6, 67%). The local responders perceived significantly more health problems than the international responders (local responders, 61%; international responders, 43%; *χ*^2^ test, *p* = 0.001). However, no significant difference was found regardless of whether the responder was deployed on a voluntary or mandatory basis (*p* = 0.414). Among the study participants (*N* = 525), the most commonly reported health problems during field mission were feeling scared or unsafe (*n* = 100, 19%), sleeping problems (*n* = 93, 18%), and headache (*n* = 75, 14%; see Table 2). Within a week after the mission, feeling depressed (*n* = 74, 14%), fatigue (*n* = 44, 8%), and sleeping problems (*n* = 42, 8%) were the most frequently reported health problems. More than a week after the end of the mission, most responders reported that the problems had been alleviated. The health issues that persisted were mainly mental health problems such as feeling depressed, sleeping problems, and somatic issues, indicating infection (see Table 2). Addiction problems were reported with low prevalence rates, but 7% of the data were missing, compared to 5% or less for all other questions.

**Table 2:** Type of health problems reported during and after the conclusion of the field mission (*N* = 525)

	During field mission, *n* (%)	Within a week after the conclusion of the mission, *n* (%)	More than a week after the conclusion of the mission, *n* (%)
**Somatic health problems**			
Musculoskeletal injuries or pains	45 (8)	7 (1)	5 (1)
Head injury	6 (1)	0	0
Cuts or wounds	57 (11)	0	0
Burn injuries	0	0	0
Cardiovascular diseases	7 (1)	5 (1)	5 (1)
Fever/infection	18 (3)	30 (6)	9 (2)
Respiratory problems	51 (10)	6 (1)	0
Headache	75 (14)	15 (3)	3 (0)
Skin problems	18 (3)	3 (0)	0
Dizziness	42 (8)	12 (2)	0
Gastrointestinal problems	45 (9)	21 (4)	0
Dehydration	35 (7)	0	0
**Mental health problems**			
Fatigue	31 (6)	44 (8)	11 (2)
Anxiety	11 (2)	15 (3)	7 (1)
Feeling scared or unsafe	100 (19)	20 (4)	0
Sleeping problems	93 (18)	42 (8)	24 (5)
Feeling depressed	12 (2)	74 (14)	55 (11)
Addiction problems**	3 (0)	6 (1)	3 (0)

*Missing values of < 5% are not reported. **Missing data of 7%.

The female responders reported more health problems (*n* = 89, 54%) than the male responders (*n* = 155, 43%; *χ*^2^ test, *p* = 0.048). Female reported more anxiety (female *n* = 24, 15%, male *n* = 9, 15%, *χ*^2^ test, *p* = 0.001), more sleeping problems (female *n* = 37, 22%, male *n* = 56, 18%, *χ*^2^ test, *p* = 0.013) and more frequently reported feeling blue or depressed (female *n* = 63, 38%, male *n* = 78, 22%, *χ*^2^ test, *p* = 0.001) compared to male responders. No significant difference in the incidence rates of health problems was found between the married and single responders or between the responders with and without children. No significant difference could be seen between experienced responders and first-time responders nor depending on the age of the responder or type of response.

### Medical care during field mission, medical evacuations, or unplanned conclusion of the mission

Approximately 14% of all who reported health problems received professional care in the field. The five most common reasons for receiving medical treatment without being evacuated were head injuries with wounds, headache, fever, anxiety, dizziness, and dehydration. The male responders were more likely to seek professional medical care during the mission (20% vs. 3%; *χ*^2^ test, *p* = 0.042). The study participants with no previous experience from disaster deployments were associated with seeking medical support during the mission (first mission, 17%; previous experience, 7%; *p* = 0.038).

In total, 18 study participants (3%), including 15 men and 3 women, were evacuated or terminated their field mission due to medical reasons. Their mean age was 46 years and did not significantly differ from that of the study participants who were not evacuated (*p* = 0.326). The reasons for medevac/unplanned conclusion of mission were a combination of fever and respiratory problems (*n* = 4), cardiovascular problems with or without respiratory problems (*n* = 7), musculoskeletal problems (*n* = 2), and unknown reasons (*n* = 5). Gender, preparatory training on health issues, length of mission, or previous mission experiences was not associated with medical evacuation (logistics regression for medical evacuation as outcome: *R*_2_ = 0.009, *p* = 0.331).

### Overall health after deployment to the earthquake disaster area

The mean (SD) EQ-VAS score for overall health after the mission based on the survey responses was 75 (16) (range, 32–100). Several respondents (*n* = 303, 56%) reported that their health status was the same as that before their deployment, whereas some (*n* = 122, 24%) had better health afterward than before, and others (*n* = 99, 18%) reported worse health status upon the conclusion of the mission. After the mission, approximately 58 (11%) and 44 (8%) of all study participants could not return to work within a week and after a week or more, respectively. The most frequently reported reasons for this were sleeping problems (*n* = 11), feeling depressed (*n* = 11), fatigue or anxiety (*n* = 6), gastrointestinal issues (*n* = 6), fever/infection (*n* = 3), dizziness (*n* = 3), and cardiovascular disease (*n* = 2; one study participant reported several conditions).

### Pre-deployment health training and follow-up health checkups

A total of 41% (*n* = 213) of the responders had received pre-deployment training on health risks during missions. Such training was not associated with the occurrence of health problems during or after the mission (study participants with health problems and pre-deployment health training compared with those health problems without training: 42% vs. 66%, *p =* 0.210). However, a statistically significant inverse association was found between training and medical evacuation from the field (respondents with preparatory training with evacuation compared with those with training without evacuation: 9% vs. 3%, p = ≤ 0.001).

Half of the study sample (*n* = 270, 50%) reported that their employer offered health examinations and follow-up upon completion of the mission, whereas 35% (*n* = 192) were not offered any health follow-up, and 11% (*n* = 57) did not know if their employer offered such services.

## Discussion

The disaster responders reported a significant impact on both mental and physical health both during and after the mission. Serious conditions that led to evacuation or unplanned end of mission were reported in a few responders. However, some health problems that were reported persisted more than a week after the end of the mission.

Health outcomes among disaster responders are likely poorly monitored and underreported [7]. Therefore, it is surprising that half of the study sample in this study reported health problems related to the mission. Both local and international responders might be faced with challenges such as a dangerous disaster environment, spartan working and living conditions, socioeconomic and cultural factors, and exposure to human suffering that directly or indirectly cause physical and mental health problems to disaster responders [14]. In this study, the local responders reported significantly more health problems than did the international responders. This is expected because the level of personal exposure might be higher, and returning to an unaffected context is not possible for local responders. Being a local responder may entail being both a victim and a helper at the same time [15]. Considering that the impact of the recovery process, both for the affected individuals and the community, will remain over a long time after international responders have left, the well-being of local responders is extremely important for overall society resilience.

Mental health issues were reported among quite a large number of responders and appeared to be more persistent than the reported physical health problems. Disaster responders may be exposed to overwhelming impressions and extreme and long-lasting stress, great human suffering, potentially traumatic events, and emotionally draining events such as violence, separation, or grief. In addition, working conditions might imply a high rate of unpredictability, unsafe environment, long working days, and spartan working and living conditions [16, 17]. In addition, being deployed to a disaster area entails a huge amount of uncertainty, which has been reported to be the essence of stress [18]. When good intentions and altruistic motives to respond to a disaster despite risks may not be met or needed, moral stress and negative feelings might occur [7, 19]. Female responders reported more mental health problems compared to male responders. Previous meta-analysis on the prevalence of post traumatic stress (PTSD) among disaster responders found no gender differences, but an increased prevalence among personnel deployed within the emergency medical services [20]. Other studies suggest that working close to patients, such as being a nurse or in the emergency medical services, increased the risk for anxiety or depressive symptoms rather than the gender [21]. However, no correlation between types of response and mental health problems was detected in this study. It should also be noted that in most studies on disaster responders, female participants are a minority [20]. In this survey, no deeper explanations or perceived cases for the occurrence of mental health problems were sought, but to fully understand the processes that led to perceived feelings of being depressed or other mental health problems, such studies are needed.

Pre-deployment assessment of personal traumas and psychiatric history and length of employment, exposure to traumatic events, emotional involvement, perception of risks, and social support has been found to influence the mental well-being of disaster responders [22]. Given this complexity, both individual and organizational strategies are necessary to foster psychological resilience among disaster responders [6, 22]. Pre-deployment screening and selection processes and pre-deployment training on stress management and psychological first aid have been suggested to reduce mental health problems among disaster responders [18]. During the operation, good leadership and social support within the team are important to mitigate stress [20, 23]. Moreover, screening for mental health problems after mission conclusion and offering professional support to those with an increased risk of long-term mental health problems have been recommended [18, 22]. However, despite the accumulating literature on mental health risks and mitigating factors, no universal consensus has been reached on the methods or strategies to prepare or support disaster responders for their missions [16, 22–24].

The concept of duty of care, which is a legal or moral obligation to ensure the safety or well-being of others that most often applies to employers caring for employees [25], is highly relevant for disaster responders [26, 27]. One aspect of the duty of care is to manage health problems in the field [14]. This study shows that such medical support should provide medical care covering injuries, non-trauma conditions, and mental health problems. Pre-deployment prevention measures include risk assessments, pre-deployment health checkups, specific training to avoid health problems during and after deployment, strategies to organize the work to minimize health problems, medical checkups, and vaccinations [14, 27]. This study shows that pre-deployment training on how to mitigate health risks did not decrease the incidence of perceived health problems but minimized the risk of serious conditions that led to medical evacuation. Therefore, it supports the idea that pre-deployment training is essential to reduce health risks and promote recovery after disaster response missions [14]. However, despite training and pre-deployment preparations, aid workers felt insufficiently prepared [27]. Few studies have focused on effective methods to prepare disaster responders for the demands from being deployed as a disaster responder [6]. This is an important focus for future research initiatives.

As some health problems were long-lasting and led to reduced ability to return to ordinary work due to medical conditions related to the mission, the question on the duty of care for individuals who respond to disasters as part of a temporary deployment must be raised. In line with previous studies [28, 29], some participants in this study reported worsened health after returning home. While knowledge on long-lasting mental health problems is covered to some extent knowledge on persistent physical health problems appears to be less studied [23, 26].

### Limitations

This study has several limitations. The study relied on a nonprobability study sample. This is a common sampling strategy in disaster research that has both advantages and limitations [30]. The self-selected study sample may come with an increased risk of overreporting, compared to randomized sample [30] and are hard to generalize. On the other hand, non-randomized study samples enable studying of populations and in situations where randomization is not possible, given ethical, safety or practical circumstances [31]. As the entire study target population could not be identified or organized for randomization, nonprobability sampling was the only sampling method considered useful for conducting this study. Also, the sample size was, from a disaster research perspective, quite large, compared to a median of 150 study participants in general disaster population studies [32]. However, the lack of baseline data is also a major limitation and should be taken into consideration when interpreting the results. To protect the identities of the study participants, no information on nationality was asked. The survey was provided in English or Turkish. However, given the languages used in free-text answers, many different languages could be identified, indicating an international study sample, even if all the study participants used the English version of the survey. It is important to highlight that most study participants in this survey were international responders. However, the largest group of disaster responders were not international personnel but national professional responders and local volunteers. As an example, more than 9,000 volunteers and staff from the Syrian Arab Red Crescent and Turkish Red Crescent were deployed after the earthquake [33]. Therefore, the study sample might not be representative of such responders but highlighted the need to further investigate whether health risks for voluntary and professional responders differ. Another limitation is the lack of baseline health data before the mission, such as the presence of chronic diseases or risk factors for the development of cardiovascular illness. These limitations make it difficult to draw general conclusions, and the extent of the generalizability of the results is unclear.

## Conclusion

Physical and mental health problems are commonly perceived among disaster responders and may cause long-term health problems among responders and reduce their effectiveness in response operations. Both individual responders and employers must be aware of such health risks. To mitigate disaster responders’ health problems and preserve their well-being, employers and sending organizations should implement pre-deployment training and provide sufficient medical and psychosocial support both during and after disaster response missions to ensure the duty of care of disaster responders.

## Electronic supplementary material

Below is the link to the electronic supplementary material.

Supplementary Material 1

## Data Availability

The datasets analysed during the current study are available from the corresponding author on reasonable request.

## Abbreviations

EMT: Emergency medical team
INSARAAG: International Search and Rescue Advisory Group
MSF: Médecins Sans Frontières
PTSD: Posttraumatic stress disorder
UN: United Nations
USAR: Urban search and rescue
UNOCHA: United Nations Office for the Coordination of Humanitarian Affairs
WFP: World Food Program

## References

[1] Emergency Response Coordination Centre (ERCC). DG ECHO Daily Map 13/02/2023. DG ECHO. 2023. https://erccportal.jrc.ec.europa.eu/ECHO-Products/Maps/Maps-Old/Daily-maps. Accessed November 21, 2023.

[2] United Nations Office for the Coordination of Humanitarian Affairs. Türkiye earthquakes urban search and rescue (USAR) team snapshot. Geneva: United Nations Office for the Coordination of Humanitarian Affairs; 2023.

[3] Guisolan SC, Ambrogi M, Meeussen A, et al. Health and security risks of humanitarian aid workers during field missions: experience of the International Red Cross. Travel Med Infect Dis. 2022;46:102275. 10.1016/j.tmaid.2022.102275. 35131427 10.1016/j.tmaid.2022.102275

[4] Bhandari D, Pandey P. Health problems while working as a volunteer or humanitarian aid worker in post-earthquake Nepal. J Nepal Med Assoc. 2018;56(211):691–5. PMC8997265 30381767

[5] Petinaux B, Macintyre AG, Barbera JA. Confined space medicine and the medical management of complex rescues: a case series. Disaster Med Public Health Prep. 2014;8(1):20–9. 10.1017/dmp.2014.1. 24528883 10.1017/dmp.2014.1

[6] Zahos H, Crilly J, Ranse J. Psychosocial problems and support for disaster medical assistance team members in the preparedness, response and recovery phases of natural hazards resulting in disasters: a scoping review. Australas Emerg Care. 2022;25(3):259–66. 10.1016/j.auec.2021.12.005. 35550349 10.1016/j.auec.2021.12.005

[7] Garbern SC, Ebbeling LG, Bartels SA. A systematic review of health outcomes among disaster and humanitarian responders. Prehosp Disaster Med. 2016;31(6):635–42. 10.1017/s1049023x16000832. 27641075 10.1017/S1049023X16000832

[8] Khatri KJ, Fitzgerald G, Poudyal Chhetri MB. Health risks and challenges in earthquake responders in Nepal: a qualitative research. Prehosp Disaster Med. 2019;34(3):274–81. 10.1017/s1049023x19004370. 31204642 10.1017/S1049023X19004370

[9] Hugelius K, Adolfsson A. The HOPE model for disaster nursing: a systematic literature review. Int Emerg Nurs. 2019;45:1–9. 10.1016/j.ienj.2019.03.007. 31005569 10.1016/j.ienj.2019.03.007

[10] Harrell M, Selvaraj SA, Edgar M. Danger! Crisis Health workers at Risk. Int J Environ Res Public Health. 2020;17(15). 10.3390/ijerph17155270. 10.3390/ijerph17155270 PMC7432711 32707800

[11] Peytremann I, Baduraux M, O’Donovan S, et al. Medical evacuations and fatalities of United Nations High Commissioner for refugees field employees. J Travel Med. 2001;8(3):117–21. 10.2310/7060.2001.24438. 11468112 10.2310/7060.2001.24438

[12] Gjerland A, Pedersen MJ, Ekeberg O, et al. Sick-leave and help seeking among rescue workers after the terror attacks in Norway, 2011. Int J Emerg. 2015;8(1):81. 10.1186/s12245-015-0081-4 PMC4539308 26283071

[13] Westman A, Kurland L, Hugelius K. Valued technical and non-technical skills among disaster responders: a cross sectional study of disaster responders involved in the earthquake in Türkiye and Syria January 2023. BMC Emerg Med. 2024;24(1):171. 10.1186/s12873-024-01083-x. 39313809 10.1186/s12873-024-01083-x PMC11421104

[14] Khatri J, Fitzgerald G, Poudyal Chhetri MB. Health risks in disaster responders: a conceptual framework. Prehosp Disaster Med. 2019;34(2):209–16. 10.1017/s1049023x19000141. 31046867 10.1017/S1049023X19000141

[15] Hugelius K, Adolfsson A, Ortenwall P, et al. Being both helpers and victims: health professionals’ experiences of working during a natural disaster. Prehosp Disaster Med. 2017;32(2):117–23. 10.1017/S1049023X16001412. 28043240 10.1017/S1049023X16001412

[16] Kang P, Lv Y, Hao L, et al. Psychological consequences and quality of life among medical rescuers who responded to the 2010 Yushu earthquake: a neglected problem. Psychiatry Res. 2015;230(2):517–23. 10.1016/j.psychres.2015.09.047. 26476590 10.1016/j.psychres.2015.09.047

[17] Mao X, Fung OWM, Hu X, et al. Psychological impacts of disaster on rescue workers: a review of the literature. Int J Disaster Risk Reduct. 2018;27:602–17. 10.1016/j.ijdrr.2017.10.020.

[18] Peters A, McEwen BS, Friston K. Uncertainty and stress: why it causes diseases and how it is mastered by the brain. Prog Neurobiolog. 2017;156:164–88. 10.1016/j.pneurobio.2017.05.004. 10.1016/j.pneurobio.2017.05.004 28576664

[19] Southwick SM, Charney DS. Resilience. The science of mastering life’s greatest challenges. Second edition. New York: Cambridge University Press; 2018.

[20] Martínez A, Blanch A. Are rescue workers still at risk? A meta-regression analysis of the worldwide prevalence of post-traumatic stress disorder and risk factors. Stress Health. 2024;40(4):e3372. 10.1002/smi.3372. 38217850 10.1002/smi.3372

[21] Hill JE, Harris C, Danielle LC, Boland P, Doherty AJ, Benedetto V, Gita BE, Clegg AJ. The prevalence of mental health conditions in healthcare workers during and after a pandemic: systematic review and meta-analysis. J Adv Nurs. 2022;78(6):1551–73. 10.1111/jan.15175. 35150151 10.1111/jan.15175 PMC9111784

[22] Brooks SK, Dunn R, Amlôt R, et al. Social and occupational factors associated with psychological distress and disorder among disaster responders: a systematic review. BMC Psychol. 2016;4:18. 10.1186/s40359-016-0120-9. 27114240 10.1186/s40359-016-0120-9 PMC4845476

[23] Bonanno GA, Westphal M, Mancini AD. Resilience to loss and potential trauma. Rev Annu Rev Clin Psychol. 2011;7:511–35. 10.1146/annurev-clinpsy-032210-104526. 21091190 10.1146/annurev-clinpsy-032210-104526

[24] Berger W, Coutinho ES, Figueira I, et al. Rescuers at risk: a systematic review and meta-regression analysis of the worldwide current prevalence and correlates of PTSD in rescue workers. Soc Psychiatry Psychiatr Epidemiol. 2012;47(6):1001–11. 10.1007/s00127-011-0408-2. 21681455 10.1007/s00127-011-0408-2 PMC3974968

[25] Godkin D, Markwell H. The duty to care of healthcare professionals: ethical issues and guidelines for policy development. Toronto, Canada: Joint Center for Bioethics, University of Toronto; 2003.

[26] Quevillon RP, Gray BL, Erickson SE, et al. Helping the helpers: assisting staff and volunteer workers before, during, and after disaster relief operations. J Clin Psychol. 2016;72(12):1348–63. 27505124 10.1002/jclp.22336

[27] McDiarmid M, Crestani R. Duty of care and health worker protections in the age of Ebola: lessons from Médecins sans Frontières. BMJ Global Health. 2019;4(4):e001593. 10.1136/bmjgh-2019-001593. 31543992 10.1136/bmjgh-2019-001593 PMC6730573

[28] Costa M, Oberholzer-Riss M, Hatz C, et al. Pre-travel health advice guidelines for humanitarian workers: a systematic review. Travel Med Infect Dis. 2015;13(6):449–65. 10.1016/j.tmaid.2015.11.006. 26701861 10.1016/j.tmaid.2015.11.006

[29] Dahlgren AL, DeRoo L, Avril J, et al. Health risks and risk taking behaviors among the International Committee of Red Cross (ICRC) expatriates returning from humanitarian missions. J Travel Med. 2009;16:382–90. 19930377 10.1111/j.1708-8305.2009.00350.x

[30] Stratton SJ. Data sampling strategies for disaster and emergency health research. Prehosp Disaster Med. 2019;34(3):227–9. 10.1017/s1049023x19004412. 31204646 10.1017/S1049023X19004412

[31] Vahedi L, Qushua N, Seff I, Doering M, Stoll C, Bartels SA, Stark L. Methodological and ethical implications of using Remote Data Collection Tools to measure sexual and Reproductive Health and gender-based violence outcomes among women and girls in Humanitarian and Fragile settings: a mixed methods systematic review of peer-reviewed research. Trauma Violence Abuse. 2023;24(4):2498–529. 35607868 10.1177/15248380221097439 PMC10486180

[32] Norris FH. Disaster Research methods: past progress and future directions. J Trauma Stress. 2006;19(2):173–84. 16612819 10.1002/jts.20109

[33] American Red Cross, Cross R. Red Crescent teams responding to earthquake in Türkiye and Syria, 1 March 2023, 2023. https://www.redcross.org/about-us/news-and-events/news/2023/red-crescent-teams-responding-to-earthquake-in-turkey-and-syria.html. Accessed December 21, 2023.

