# Supplementary material for: Health problems among disaster responders to the 2023 Turkey-Syria earthquake: a cross-sectional study
Source: BMC Emerg Med. 2024 Dec 2;24:226. doi: 10.1186/s12873-024-01143-2 (PMC11610220; doi:10.1186/s12873-024-01143-2)
Supplement: Supplementary file 1 — Supplementary Material 1 [file 12873_2024_1143_MOESM1_ESM.docx]

Supplementary file: Questionnaire

| **Question** | **Answer alternatives** |
| --- | --- |
| Are you… | Female  Male  Other  I don't want to answer this question |
| How old are you? (in years) | (Number) |
| What is your marital status? | Single (or divorced/separated)  Married or in a domestic partnership  Widowed  Other |
| Do you have children? | No  Yes |
| What is your profession? (in your everyday work) | Medical doctor/physician  Nurse  Medic/paramedic  Rescuer/firefighter  Building engineer  Police officer/military professional  Social work/psychologist  Logistic officer  Information and communication technology officer  Water and sanitation expert  Humanitarian aid worker  Other (please comment below) |
| Did you participate in the response | As part of your ordinary work (e.g., as a nurse or firefighter working every day in the disaster area)  As a local responder in a temporary deployment (e.g., as part of a local medical team deployed to the disaster area)  As an international staff (e.g., as part of an international team) |
| Did you participate in the response as… | Part of your ordinary work or mandatory deployment  Part of your ordinary work or voluntary deployment  Part of your temporary or mandatory deployment  Part of your temporary or voluntary deployment |
| How many disaster response missions have you been deployed in (including this)? | (Number) |
| What was your function/position during this mission? | Emergency medical team (EMT)  Health- or medical (not EMT)  Mental health and psychosocial support  Urban search and rescue (USAR)  Needs assessment  Management, coordination, and logistics (e.g., UNDAC or EUCPT)  Shelter  Food or nutrition  Water and sanitation  Early recovery  Other humanitarian aid  Other |
| How long was your deployment/mission? | 1–7 days  8–21 days  More than 21 days |
| Have you done any preparatory training on health risks or how to stay healthy in disaster response missions? | Yes  No  I don't know |
| Finally, we would like to ask a few questions about your health during and after your mission. Did you experience any of the below health issues during or after your mission? (Multiple markings can be made.)  Musculoskeletal pains or injuries (e.g., back pain, fractures, or other injuries)  Head injury  Cuts or wounds  Burn injuries  Cardiovascular diseases (e.g., chest pains, heart attack, and stroke)  Fever or infection disease  Respiratory problems  Headache  Skin problems  Dizziness  Gastrointestinal issues (abdominal pain, diarrhea, and nausea)  Dehydration  Fatigue  Anxiety  Feeling scared or unsafe  Sleeping problems  Feeling depressed  Addiction problems (e.g., increased use of alcohol)  Other physical health issues  Other mental health issues | For each subquestion:  No  Yes, during the field mission  Yes, within a week after the end of the mission  Yes, a week or longer after the end of the mission |
| Did this health issue require | For each subquestion:  Self-care  Professional medical care or psychosocial counseling during the mission/disaster response in the field  Medical evacuation from the field (or unplanned end of the mission)  Professional medical care or psychosocial counseling after the mission (other than routine health checkup) |
| Were you unable to return to your ordinary job due to health issues that arose during your temporary mission? | Yes, for less than 7 days  Yes, for more than 7 days  No |
| Have you sought professional support (e.g., counseling) for stress or other mental health issues related to the disaster response? | Yes  No, I don't have a need for it  No, but I think I would benefit from such support |
| Does your organization/employer have routines for following up on your health after being deployed in a disaster? | Yes  No  I don't know |
| In general, how would you rate your current health if 0 is very bad and 100 is excellent? | Number from 0 to 100 |
| How would you assess your overall health after the mission compared with before the mission/disaster? | My health is the same after as before  My health is better after than before  My health is worse after than before |
| Do you have any other thoughts you would like to share with the research team? | Free text answer |
